# The Impact of a Brief Psycho-Education and Skills Intervention on Trauma Symptoms in a Prison Setting: Proof of Concept Using Group and Case Analysis

**DOI:** 10.1177/0306624X251405449

**Published:** 2025-12-28

**Authors:** Maia Winsor, Matthew Gobbett, Jason Davies

**Affiliations:** 1 Swansea University, UK; 2 Forensic Psychology Consultancy (UK) Ltd, Cardiff, UK

**Keywords:** trauma, intervention, psycho-education, case series, stabilization

## Abstract

The links between trauma and poorer physical health and psychological well-being in adulthood are now well established. Research shows levels of trauma are especially high amongst those who are incarcerated although evidence on what works to help this group is limited. To examine the effectiveness and acceptability of participation in a new brief, structured group intervention in fostering stabilization through reducing the symptoms of post-traumatic stress disorder (PTSD) and improving mental health in male inmates who have experienced trauma. Participants completed questionnaire measures before and after treatment and provided feedback via semi structured interviews. Quantitative data were analyzed at the group and individual level and qualitative data were analyzed using content analysis. At the group level, participants showed a significant improvement in scores for PTSD, anxiety and general mental health. Examination of individual scores showed reliable and clinically meaningful change for some individuals in trauma and mental health symptoms with little change observed for wellbeing and resilience. Participants feedback was generally positive with suggestions for future changes. Engaging in a brief group-based trauma intervention was acceptable to participants and offered significant improvements mental health and PTSD for some incarcerated individuals who have experienced trauma. This intervention may provide a cost effective and resource light approach to phase 1 PTSD treatment ahead of phase 2 treatment. It would be valuable for future research to develop understanding on who the intervention is most effective for.

## Plain Language Summary

A short group program to help prisoners cope with trauma: findings from a first proof of concept study.

People in prison often have a history of trauma, which can lead to serious mental health problems. This pilot study tested a group program which was delivered over 4 sessions. The intervention was designed to help male prisoners who have experienced trauma develop skills to help better manage their mood and trauma symptoms. The study measured overall changes across the group of participants and reviewed each individual to determine what, if any, changes were experienced. Those who participated were also interviewed about their experience of the group. The results showed that the program helped reduce symptoms of post-traumatic stress (PTSD) and anxiety for the participant group as a whole. Some individuals reported large improvements, while others had smaller to no changes. No participants reported getting worse as a result of the program. Most participants said they found the program helpful and had ideas for how it could be further improved. This proof of concept study shows that the intervention is worthy of more detailed research and evaluation. Overall, this kind of group support could be a useful and low-cost way to help people in prison start to recover from trauma.

## Introduction

Trauma is a complex and multi-faceted concept encompassing both exposure to distressing or life-threatening events and the subsequent psychological and physiological sequelae. Diagnostic frameworks such as DSM-5 (American Psychiatric Association [APA], 2022) and ICD-11 (WHO, 2022) highlight this distinction, defining trauma-related disorders as arising from but not synonymous with the traumatic events themselves, whilst the Substance Abuse and Mental Health Services Administration (Substance Abuse and Mental Health Services Administration [SAMHSA], 2014) describe trauma as an emotional response to a distressing or life-threatening event, or series of events. Events that may be traumatic for individuals include natural disasters, violent acts such as terror attacks, physical or sexual abuse, accidents or witnessing violence (APA, 2022; Brassett & Vaughan-Williams, 2012; National Institute of Mental Health (NIMH), 2024). ICD-11 further differentiates between post-traumatic stress disorder (PTSD) and complex PTSD (CPTSD), the latter reflecting pervasive and enduring disturbances in self-organization (e.g., affect dysregulation, negative self-concept, relational difficulties) that often result from sustained or repeated interpersonal trauma (Cloitre et al., 2012). Adverse Childhood Experiences (ACEs) represent a subset of traumatic environmental stressors, such as living in a household that is impacted by domestic violence or substance abuse, as well as childhood abuse and neglect (Bellis et al., 2018). Such experiences have been shown to occur frequently in the general population; for example, in a survey of 2,028 adults, 47% of adults reported experiencing at least one ACE and 14% had experienced four or more (Bellis et al., 2016).

The sequelae of ACEs and trauma experiences can be long lasting and wide-ranging. Childhood exposure has been linked to dysregulation of cardiovascular, immune, and endocrine systems (Shonkoff et al., 2012), as well as heightened risk of depression, anxiety, PTSD, and other psychiatric disorders (Abate et al., 2025). Evidence indicates a dose–response effect, with cumulative ACEs associated with chronic disease, health-risk behaviors, and poorer mental health outcomes in adulthood (Felitti et al., 1998; Hamby et al., 2021).

Within the prison context, whilst Trauma prevalence rates vary by data collection method and country, a pooled point prevalence rate for PTSD of 6.2% has been reported in male prisoners (based on 50 samples from 20 countries; Baranyi et al., 2018). A recent UK study found a slightly higher male point prevalent rate of 7.7% for PTSD with a rate of 16.7% for CPTSD (Facer-Irwin et al., 2022). It is also important to note that those who are incarcerated frequently experience potentially traumatic events within prison and that such experiences may themselves be associated with subsequent PTSD (Piper & Berle, 2019). In response to such evidence, recommendations have been developed to improve the identification, treatment and support for individuals who have experienced trauma and are living in prison (Crole-Rees et al., 2026). These recognize the need to improve detection rates and to provide interventions that range from trauma-informed psychoeducation and stabilization-based approaches through to trauma focused interventions. Additionally, it is argued that such pathways of assessment and response should be situated within prison organizations and staff teams that are trauma informed in order to avoid repeating, triggering or generating new trauma experiences within the prison context (Davies & Jones, 2024).

Where treatment is to be provided, both the triphasic model, which provides a sequenced and structured approach to the treatment of trauma (Herman, 2015) and the two phase approach to trauma treatment have been widely adopted. In both approaches phase 1 concerns stabilization, safety and psychoeducation and phase 2 provides interventions designed for trauma processing; the triphasic model adds a third phase of reintegration, reconnection, and recovery (McFetridge et al., 2017). Proponents of these approaches argue that stabilization work is necessary to establish psychological safety upon which trauma focused interventions can be provided. Within a prison context, phase 1 treatments are typically delivered in a group context whilst phase 2 interventions may be group or individual (with individual phase 2 interventions being more effective; Malik et al., 2023). The evidence suggests that whilst phase 1 interventions can lead to positive symptom change in and of themselves, the level of change is significantly greater in phase 2 individual interventions (Malik et al., 2023). Consequently, attempts to design comprehensive structured programs for addressing trauma symptoms amongst those in criminal justice settings have been described (e.g., the RISE program (Renn, Tamburri et al., 2025) and the STAIR intervention (Renn, Herod et al., 2025; Tripodi et al., 2022). These approaches have been designed for specific groups and are yet to be widely evaluated.

While phase-based approaches are common, critics argue that the presence of phase 1 may delay or deny access to effective trauma-focused (phase 2) treatments (De Jongh et al., 2016) or that phase 1 is not necessary. Outcome research in this area is mixed, possibly the result of the different inclusion criteria for study engagement. For example, a systematic review and meta analysis by Willis et al. (2023) concluded that phased approaches were associated with large treatment effects and that the group process and skills acquisition contained within the stabilization phase (phase 1) was of benefit. In contrast, a recent study with those who have PTSD due to ACEs found that a mutli-phase intervention was not cost-effective (based on PTSD remission as an outcome) when compared with a phase 2 only trauma intervention using EMDR (van Vliet et al., 2024). Whilst outcomes were comparable, the phase 2 only intervention was shorter in duration (16 sessions as opposed to 24 sessions) and thus less expensive. However, when considering the prison context it is important to note that both these studies addressed non-prison populations. Therefore when considering the arguments for a multi-phase or phase 2 only approach within a prison context it is important to recognize that specialist phase 2 interventions may not be available or easily deployed. It is also important to note that many existing phase 1 interventions delivered in prison have an extended duration (lasting 3–6 months e.g., Malik et al., 2023). Consequently, research on short duration phase 1 interventions is warranted to determine whether such approaches may reduce distress in a prison context. If effective, such interventions may be delivered as a foundation for stage 2 treatment (i.e., in a multi-phase intervention) or when resources and phase 2 treatment availability are limited and safety, stability and readiness for trauma-focused therapy are uncertain.

This study will examine a newly developed, brief, group based psychoeducational program developed to facilitate safety and stabilization for people in prison who have experienced trauma. The group was developed to address a gap in service provision for a phase 1 structured program with minimal resource demands that can be delivered in a short timeframe. BASES (Becoming Aware and Safely Exploring Symptoms of trauma; Forensic Psychology Consultancy, 2024), draws on Compassion Focused Therapy (Clark et al., 2014) and Positive Psychology (Seligman & Csikszentmihalyi, 2000) to develop understanding of experiences, normalize responses and foster skills to assist with the short-term management of trauma related symptoms. Participants can also opt into a journaling component which is based upon the basic writing paradigm (Pennebaker, 1997) and undertaken immediately prior to group sessions 2, 3, and 4.

Recent guidance on behalf of the Medical Research Council emphasizes the importance of a phased approach to treatment evaluation, using theory, feasibility testing, and research to examining factors such as the acceptability and deliverability of the treatment alongside its effectiveness (Skivington et al., 2021, p. 2). As a proof of concept study for a new brief intervention, a mixed methods approach will be adopted combining group level and case series analysis (Davies et al., 2007) alongside structured participant interviews. These will examine self-reported wellbeing, mental health and PTSD symptoms of individuals in a prison setting and the acceptability of the intervention.

It is hypothesized that BASES will be acceptable to participants and, at a group level, participation in the intervention will positively impact self-reported PTSD symptoms, mental health and well-being (within group analysis). Individual level analysis will be used to examine the nature and range of intervention impact and to identify any individuals who experience iatrogenic effects (e.g., drop out; worsening of self reported symptoms).

## Method

### Ethics

Ethical approval was obtained from Swansea University’s School of Psychology ethics committee (Ref: 2 2024 8971 8322) and the HMPPS National Research Committee (Ref: 2024-062).

### Intervention

The BASES intervention (Forensic Psychology Consultancy, 2024) is a 4 hr (1 hr per week) psycho-education group delivered by a team of two to three facilitators. The group is designed for those who experience symptoms consistent with PTSD/CPTSD with a focus on safety and stabilization (phase 1) of trauma recovery. The intervention adopts an ‘in the moment’ stance to support individuals with managing difficulties they are experiencing at present relating to previous traumatic events. Each session combines specific trauma knowledge with skills development as outlined in the manualized curriculum:

- session 1 – safety and stabilization (introduction to the phased approach to trauma recovery, trauma responses, importance of safety, compassion, grounding techniques and mindfulness)
- session 2 – building your safety toolkit (basic neurobiological explanation of trauma, explanation of the Window of Tolerance trauma triggers and skills for hyper and hypo arousal)
- session 3 – improving your sleep (overview of sleep problems & nightmares, nighttime routine, progressive muscle relaxation techniques)
- session 4 – moving on (practicing self soothe and guided mindfulness, self care and the future).

Participants are encouraged to practice skills learned between sessions and to feedback and discuss skills use experience at the start of each session. The intervention is designed to promote mood stability and build skills in coping with trauma symptoms.

### Participants

All nine participants were male, aged between 18 and 55 years and had been in prison at least twice (see Table 1). Most participants (*n* = 7) opted into the additional journaling component prior to sessions 2, 3, and 4 (participants 1 & 6 chose not to complete this component). Current sentence length ranged from 2 to 13 years and time already spent in prison for the sentence varied from 8 months to over 6 years. The time since the initial trauma experience was 2.5 to 37 years. Those experiencing current symptoms consistent with PTSD who were referred to the Dyfodol psychology services or New Pathways counseling service within the prison were invited to join the BASES pilot.

**Table 1.** Demographic Information of Participants.

Participant	Age	No. of times in prison	Length of current sentence	Time already spent	Time since trauma occurred
Person 1	36–45	6	3 years	2 years	4 years ago.
Person 2	18–25	2	8 years	1 year	10 years ago.
Person 3	26–35	10+	9 years, 8 months	6 years	9 years ago.
Person 4	36–45	2	4 years, 7 months	1.5 years	10 years ago.
Person 5	46–55	Not reported	12 years	2 years	31 years ago.
Person 6	36–45	10+	13 years	6 years 4 months	37 years ago.
Person 7	26–35	15	4 years 8 months	1 year, 6 months	2.5 years ago.
Person 8	46–55	16	6 years	5 years, 2 months	20 years ago.
Person 9	36–45	10	28 months	8 months	12 years ago.

### Materials

#### PTSD Checklist for DSM-5 (PCL-5; Weathers et al., 2013)

The PCL-5 is a 20-item assessment that measures the symptoms of PTSD such as loss of interest in previously enjoyable activities or difficulty concentrating. Participants rate their experience of each symptom on a five-point Likert scale, from ‘0 = Not at all’ to ‘4 = Extremely’. A total score, ranging from 0 to 80, is created through summing each item. A score of 32 and above indicates probable PTSD; it is suggested that a change of at least 10 points is required to determine clinically meaningful change. This questionnaire has been found to be reliable and valid self report measure of PTSD symptoms (Blevins et al., 2015).

#### The Short Warwick-Edinburgh Mental Wellbeing Scale (SWEMWBS; Stewart-Brown et al., 2009)

The SWEMWBS is comprised of seven items that measure mental well-being, such as optimism about the future or feeling relaxed. Participants rate each statement on a five-point Likert scale, from ‘1 = None of the time’ to ‘5 = All the time’. A total score is calculated by summing the score of each item and then transforming it using the SWEMWBS conversion table. Scores range from 7 to 35 with lower scores indicating poorer well-being. This questionnaire has been reported to be reliable and valid for use with a general population where the mean wellbeing score is around 23 (Koushede et al., 2019), however no data exists on its use within a custodial setting.

#### Brief Resilience Scale (BRS; Smith et al., 2008)

The BRS is a six-item measure that investigates a person ability to recover from stress. This measure asks participants to rate three positively and three negatively worded statements such as ‘I tend to bounce back quickly after hard times’, or ‘I have a hard time making it through stressful events’, on a five-point Likert scale ranging from ‘Strongly Disagree’ to ‘Strongly Agree’. A total score is obtained from calculating the mean of all six items (with negatively worded items are scored in reverse). The authors report good internal consistency and adequate test-retest reliability over 1 month with scores for ‘normal resilience’ falling between 3 and 4.3.

#### Patient Health Questionnaire (PHQ-4; Kroenke et al., 2009)

The PHQ-4 is a brief screening tool to assess an individual’s levels of anxiety and depression. Respondents are asked how frequently they have been affected by problems in the past 2 weeks, such as feeling nervous or down, on a four-point Likert scale; ranging from ‘0 = Not at all’ to ‘3 = Nearly every day’. Scores can be derived to index depression, anxiety and an overall (combined) score. Research suggests good reliability for the overall (combined) scale (Khubchandani et al., 2016), with scores of 3 or more on the depression or anxiety subscales indicative of problems in these areas.

#### Group Content and Experience – Participant Interview Schedule

Feedback on participant’s experiences of the group (including the format and delivery of the sessions) and the content (what was/was not covered) was collected via brief individual interviews guided by a bespoke interview schedule. Within this, participants were also asked to identify content or delivery aspects which they particularly liked or disliked and for any suggestions for improving the group.

### Procedure

All those who had agreed to take part in the brief intervention were invited to an introductory session in which the nature of the intervention and the purpose of the research were outlined and an information sheet about the research was provided. It was stressed that individuals could participate in the intervention without engaging in the research component, and that they could withdraw from the research component should they later change their mind. Potential participants were then provided a consent form to be completed and signed if they agreed to participate in the research. All those who attended the introductory session agreed to participate in both the intervention and the research. Prior to the first intervention session, participants were required to complete and return a questionnaire pack. Within this, participants were required to create a unique ID to allow pre intervention and post intervention questionnaires to be anonymously linked, to provide basic demographic information (e.g., age, current sentence length), and to complete the four self report measures. Participants then attended the four intervention sessions. Those who completed the optional journaling component of the intervention were asked to provide ratings at the end of each journaling episode to indicate the extent to which they ‘concentrated whilst journaling’ and ‘enjoyed the journaling process’ (both rated from 1 = ‘not al all’ to 5 = ‘extremely’). Two weeks after the final intervention session, participants attended a feedback session in which they completed the questionnaire pack, took part in a brief interview and were provided with a debrief form.

### Approach to Analysis

Quantitative data were analyzed at the group level using paired *t-*tests and individually through graphical and reliable change/clinically meaningful change methods. The interview transcripts were annotated and coded using content analysis.

## Results

### Group Level Change

Paired-samples *t*-tests were conducted to compare pre-treatment and post-treatment group level scores. No statistically significant change over time was observed for the SWEMWBS, BRS or PHQ-4 depression scores. For the PCL-5, there was a significant difference between scores at time one (*M* = 59.111, *SD* = 16.22) and time two (*M* = 49.777, *SD* = 13.572); t (8) = 2.061, *p* < .037. There was also a significant difference for PHQ-4 anxiety scores at time one (*M* = 5.000, *SD* = 1.000) and time two (*M* = 3.111, *SD* = 2.027); *t* (8) 2.639, *p* < .015 and for PHQ-4 total scores at time one (*M* = 9.667, *SD* = 1.803) and time two (*M* = 6.778, *SD* = 3.866); *t* (8) = 2.193, *p* < .030.

### Individual Change

Pre-intervention, eight out of nine participants were at least 1 s.d. below the community mean for well-being and were above the cut-off for PTSD on the PCL-5. All participants were at least 1 s.d. below the published mean for resilience and met the cut-off scores for anxiety and depression, with two scoring in the moderate and seven in the severe range for the PHQ-4 total score. Table 2 shows baseline and normative information for each dimension of the questionnaire.

**Table 2.** Baseline and Normative Data.

Measure	Person 1	Person 2	Person 3	Person 4	Person 5	Person 6	Person 7	Person 8	Person 9	Normative data^a^
SWEMWBS	17.43	14.08	14.08	16.36	17.43	14.08	24.11	18.59	16.88	23.5
PCL-5	47	69	76	62	64	30	77	66	41	<32
BRS	2.6	2.16	1	2	2	2.3	2.3	2.5	1.5	3.7
PHQ-4 depression	5	5	6	5	4	4	5	5	3	<3
PHQ-4 anxiety	5	5	6	6	5	4	6	5	3	<3
PHQ-4 total	10	10	12	11	9	8	11	10	6	<6

a Scores which indicate average community scores (SWEMBS & BRS) or non-clinical/healthy range (PCL-5, PHQ-4).

**Figure 1.**
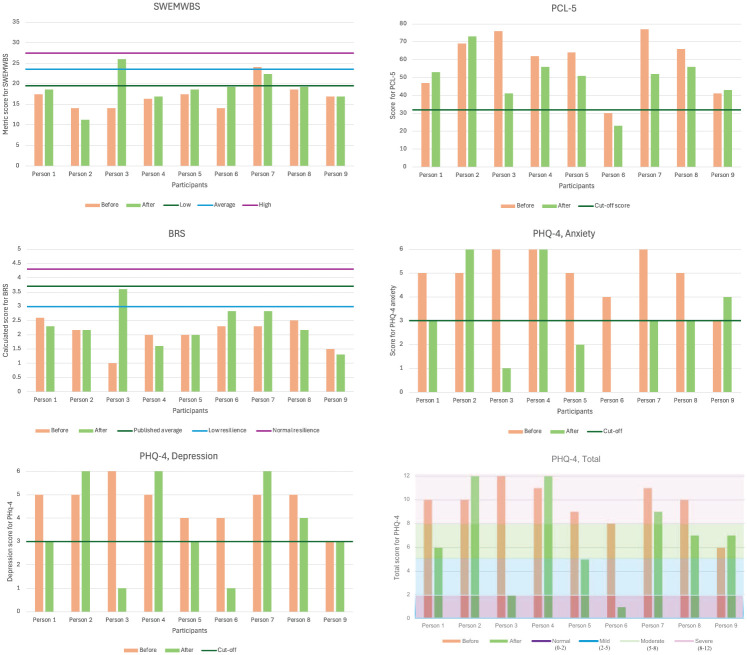
Graphical data from questionnaires.

Graphical analysis (Figure 1) showed that individual level change over time varied in direction and degree by person and by measure. To assess the extent to which the observed changes might represent significant and meaningful change, three robust tests of change were applied where applicable to the individual scores – exceeding the Reliable Change Index for the test (RCI; Morley & Dowzer, 2014) using a value of 1.96; moving across a threshold boundary (e.g., from severe to moderate); meeting criteria set for accepting change as meaningful (i.e., PCL-5 – a 10 point change). For the significant and meaningful change analysis the sub-scales of the PHQ-4 (anxiety and depression) were not included to avoid duplication from this scale.

The extent to which individual changes observed in the graphical analysis met the statistically reliable (R) and clinically meaningful (CM) threshold tests set can be seen in Table 3. No significant and/or meaningful deterioration was seen for any of the participants, although no R and/or CM change was seen for three of the participants on any of the scales – person 2, person 4, and person 9. Reliable change in one area of assessment only was seen in person 1 and reliable change with a reduction of 10 points in trauma symptoms was seen in person 7. The remaining four participants (persons 8, 6, 5, and 3) reported R and CM change in at least one domain, with three of these participants (persons 8, 5, and 3) also showing a change of 10 or more points in their trauma symptom endorsement.

**Table 3.** Individual Change Assessed Against Statistical and Clinically Meaningful Criteria^a^.

Participant	SWEMWBS	PCL-5	BRS	PHQ-4 total
Person 2	x	x	x	x
Person 4	x	x	x	x
Person 9	x	x	x	x
Person 1^b^	x	x	x	R
Person 7	x	R & 10	x	x
Person 6^b^	R	x	x	R & CM
Person 8	x	10	x	R & CM
Person 5	x	R & 10	x	R & CM
Person 3	R & CM	R & 10	R & CM	R & CM

Note. R = reliable change based on RCI; CM = clinically meaningful change (movement between thresholds); 10 = a change of 10 or more points (PCL-5 only); x = change doesn’t meet CM or R thresholds.

a To maximize clarity, the table is sequenced according to the extent of change observed rather than by participant number.

b Person 1 and Person 6 did not participate in the journaling component.

### Journaling Experience Ratings

Self-reported levels of concentration were generally high for the seven individuals who participated in this component (mean = 3.7) which contrasted with the self-reported level of enjoyment which were generally low (mean = 2.4; mode = 1).

### Participant Feedback of Experience

Participants reported joining the group for various reasons including ‘being willing to try anything’, ‘to build understanding’, and ‘to help with anxiety’. Most participants reported that they had enjoyed the group work with some reporting the group was ‘not something they would usually do but it was good’. No dislikes about being with peers were provided but ‘hearing other’s experiences’, ‘bouncing ideas off peers’, and ‘the sense of mentorship and support’ were common likes. Only one participant reported aspects of the group as unhelpful stating that it ‘brought up past experiences’ that caused ‘flashbacks and made them feel anxious’.

With regard to content, participants found the ‘knowledge on the brain and how things link to behaviour’ and the ‘skills taught’ helpful because it helped them to ‘remain calm’ and ‘handle emotions in situations’ as well as aiding them to ‘understand reasoning for behaviour to stop it in the future’. Of those who participated in the journaling element, views were split with three reporting a dislike for this element whilst four reported finding it useful.

Participants recalled a total of 16 skills and tools that were taught within the sessions with the most frequently remembered being ‘muscle relaxation’ and ‘the five senses’. Two participants reported not having used the skills outside the group sessions; one reported using them once and one described ‘using them without knowing it’. Of the remaining participants, skills were reported to have been used ‘two or three times and week’ (three participants) and ‘regularly’ or ‘daily’ (two participants).

Since completing the intervention, four participants reported their distress level was lower, whilst four believed their distress level had not changed, one of whom, reported that the lack of change they experienced ‘may be due to the prison setting and not the intervention’. The final participant stated that whilst their distress continued to vary throughout the day, they ‘manage it better since completing the intervention’. Three participants provided suggestions for possible improvements to the sessions namely: ‘adding an icebreaker to the start of each session to ease everyone in’, ‘adding a thinking skills section’, ‘providing more advice’, and ‘offering a few sessions on a one-two-one basis to allow participants to discuss their trauma to get it off your chest’.

## Discussion

This study found that participating in the brief, group based intervention to develop skills to address symptoms associated with trauma resulted in group level changes in trauma symptoms and anxiety. Individual analyses showed that almost half the group experienced symptom change that can be considered both reliable and clinically meaningful, with no individuals withdrawing from the intervention. Together these findings suggest that a brief knowledge and skills group-based trauma stabilization intervention is worthy of further study to determine the extent and duration of change and who might benefit most.

Whilst the reductions in reported trauma symptoms in this study did not fall below the suggested cut off score on the tool, given the brevity and pre-treatment stabilization focus of the intervention these results are encouraging and add to the evidence for potential effectiveness of interventions in this area. The reduction in trauma symptoms seen in this study are in keeping with the systematic review and meta analysis conducted by Malik et al. (2023) which found phase 1 interventions to be associated with symptom change. However it is worth noting that the intervention tested here is much shorter than any of the studies included within their review.

The reduction in anxiety, is somewhat expected given the overlap between PTSD symptoms and symptoms associated with mental health problems (e.g., Liu et al., 2021). This is also consistent with research with non-offender populations, where trauma focused interventions have been found to impact mental health disorders, specifically depression and anxiety (Alshahrani et al., 2022).

The lack of change observed in well-being may reflect both the wording of the items of on the scale (and their applicability to the prison setting) and the potential delay between symptom level change and an overall improvement in wellbeing (Davies et al., 2020). Similarly, with the exception of one individual, no significant changes were observed in reported resilience. Whilst resilience to stress may be related to symptoms and experience of trauma, the general lack of change in reported resilience may provide some evidence of discrimination within the intervention that is, to help identify the specific domains the intervention does and doesn’t effectively target. For example, there was no content in the intervention specifically addressing building resilience. Thus, the findings in relation to wellbeing and resilience might point toward tools which could be used as ‘control measures’ in future research – offering measurement that is stable over time, not directly targeted by the interventions and thus helps to control for regression to the mean or other extraneous reasons for change seen on the directly relevant measures. This could help bolster the credibility given to the areas of change that are observed especially where single case, case series and small n approaches are to be used to build the evidence base.

Participants generally viewed the group as acceptable and reported mostly beneficial impacts of peer discussions as has been found for other needs and in other settings (e.g., Biagianti et al., 2018; Pfeiffer et al., 2011). However, the focus of the discussions and, possibly, the group format was triggering for some, an issue that has been reported elsewhere (e.g., Larsen et al., 2016). It is possible that further pre-group preparation and discussion about these possibilities would be a helpful addition for participants before joining a group. It might also be possible to address this through introducing some individual sessions alongside the group which participants suggested would be helpful.

This proof of concept study shows that BASES may be a promising, brief intervention where a short phase 1 intervention for PTSD is warranted before phase 2 interventions are deployed or where resources are limited. However, it is important that further research is conducted to examine how well the intervention can be scaled up for wider delivery, whether the positive effects can be replicated in a study with a control group and who the intervention might be most beneficial for. It is also important to acknowledge that a focus on phase 1 interventions should not detract from promoting, providing and examining the impact of phase 2 trauma based interventions such as those identified in the NICE guidance.

### Study Limitations and Future Directions

The design of the study does not rule out the possibility that factors outside the group setting account for the changes observed. However, the absence of change across all participants and the profile of changes within and between individuals suggests that responses post intervention were not the result of a common extraneous factor or based on desirable responding. Even-so, future research may wish to make use of comparison groups or a more rigorous single case repeated measures design approach to examine this further. This latter approach could also provide more information on the process of individual change and, by collecting additional data about participant characteristics (including experiences of in prison trauma), may help to understand who the approach might be most beneficial for.

The measures used in this study were selected for their brevity and ease of use, however longer self-report measures and data from other sources (e.g., behavioral data, third party ratings) could add information and confidence to conclusions about the nature and extent of change. Additionally, including a 3 to 6 month follow up period would allow the longer-term impacts of the group to be determined whilst, following those who subsequently engage in trauma focused treatment could provide an understanding of what, if any, additional benefits are provided by a stabilization group intervention.

This research was undertaken with people housed in one wing in a single prison setting. Therefore, it would be advantageous for a replication study to be performed either within the same prison but on different wings, or in other prisons. This would provide information to further the understanding of the efficacy of the intervention.

## Conclusion

The group and case analyses reported in this study reveal the potential impact of a brief, skills-based stabilization intervention on trauma and mental health symptoms for some individuals who have experienced trauma and are in prison. The study also shows the usefulness of combining methods such as group and case analysis to begin to examine intervention impacts. Research is now needed to examine the impact of this intervention in other settings and to examine who the intervention might be most helpful for. Researchers and clinicians should also consider the ways in which single case and other case study methods might be used in forensic settings where the number of available participants may be small, interventions might be new and where recognizing the heterogeneity of individuals needs and responses is important.
